# Frequent mandatory COVID-19 testing may increase risky behavior

**DOI:** 10.1093/pnasnexus/pgac247

**Published:** 2022-11-04

**Authors:** Chian Jones Ritten, Linda Thunström, Todd Cherry, J D Wulfhorst

**Affiliations:** Department of Agricultural and Applied Economics, University of Wyoming, Laramie, WY 82071, USA; Department of Economics, University of Wyoming, Laramie, WY 82071, USA; Department of Economics, University of Wyoming, Laramie, WY 82071, USA; Department of Natural Resources and Society, University of Idaho, Moscow, ID 83844, USA

**Keywords:** mandatory disease testing, risk compensation, disease spread, COVID-19, health risks

## Abstract

Mandatory surveillance testing programs are popular policies aimed to control SARS-CoV-2 and may be considered for future epidemics. However, if people believe that testing lowers their risk of infection, such policies could increase risky behavior and may even cause increased pathogen spread. Using data from two US universities, we find that frequent mandatory testing is associated with greater participation in events linked to COVID-19 spread. Women seem to be driving this association, and mediation analyses suggest this is partly due to women’s higher perception of COVID-related health risks. Our results show the potential for adverse effects from epidemic control policies, both on average and across population subgroups. Undertaking mitigation measures to reduce such unintended consequences may therefore be important.

## Introduction

Worldwide, policies to control the spread of SARS-CoV-2 include social (physical) distancing, mandatory face masking, surveillance testing, and contact tracing. The effectiveness of testing policies in controlling a pathogen depends on the accuracy and speed of the tests, and how being tested affects people’s risky behavior. Herein, we focus on the latter and examine whether mandatory surveillance testing leads to increased risky behavior.

Mandatory testing can be effective at identifying and isolating infections. It may, however, also give people a false sense of protection, and therefore promote behaviors that increase pathogen spread. The notion that people increase their risky behavior in response to mandatory safety measures dates back over 50 years (1, 2). (This response to mandatory safety measures is often referred to as the Peltzman Effect and is closely related to predictions of risk homeostasis and moral hazard theories.) Peltzman, in his seminal paper, contends that people rationally balance competing demands of safety and risk-taking behavior, and mandatory safety measures will lead to an increase in risk taking (2). The policy implication is that such risk compensation behavior may offset the intended effect of mandatory safety measures. A large literature offers mixed empirical evidence and suggests that this risk compensation behavior varies across domains and generally offsets only part of the direct effects of safety measures (3, 4).

In the context of COVID-19 testing, this risk compensation theory suggests that if mandatory testing reduces the perceived probability of illness, an increase in COVID-related risky behavior may follow. Recent research provides some evidence that people increase risky behavior in response to facemask wearing and vaccines (5–7), but this is the first study to examine the behavioral responses to mandatory testing. Risk compensation behavior matters in this context because, given the exponential nature of viral spread, even small increases in COVID-related risky behaviors can translate to significant increases in infection.

The extent of risk compensation behavior may be amplified when tests are inaccurate, and results are delayed. TP-PCR tests (the most effective COVID-19 tests) detect at least 90% of infections within the first 5 days of symptoms but are less accurate before or after this window (8, 9). Also, results are often delayed for days because institutions outsource test analysis. People may therefore unknowingly be infected while perceiving themselves protected because of frequent testing, and thus engage in risky behavior based on that perception. Such unintended consequences may pose a particularly large threat to public health when incubation times are short, the virus is highly transmissible, and the risk of false negatives is high, such as with the Omicron variant (B.1.1.529) of the coronavirus (10, 11).

The mandatory surveillance testing programs during the COVID-19 pandemic offer a unique opportunity to examine the relationship between testing policies and COVID-related risky behavior. We use survey data (*N* = 1,274) to examine whether the test frequency of students (using TP-PCR tests) at two large US universities (University of Wyoming, UW; and University of Idaho, UI) is associated with students’ participation in events at high risk of promoting COVID-19 spread (12) during the academic fall semester of 2020. The two universities were selected based on their identical masking programs (required at all indoor events on campus), and similar student and state characteristics. [Both are land-grant universities with similar student numbers (11,829 and 10,791 at UW and UI, respectively), in similar-sized cities (population: 32,711 in Laramie, WY, and 25,702 in Moscow, ID), in states with similar political affiliations and with similar rates of SARS-CoV-2 infections at the time the survey was administered (7.01% and 7.10% of population had tested positive in Wyoming and Idaho, respectively (13))], combined with differences in testing programs. During the fall of 2020, UW’s policy was to test all on-campus undergraduate students twice weekly, while UI aimed to test a small random sample of students weekly. Our data thereby have considerable variance in exogenously determined test frequency across similar populations. The two university samples are balanced [following Imbens and Rubin (14)] for characteristics shown to affect willingness to take a COVID-19 test: political affiliation, religious affiliation, and perceived COVID-19 health risk (15).

## Results

**Fig. 1.**
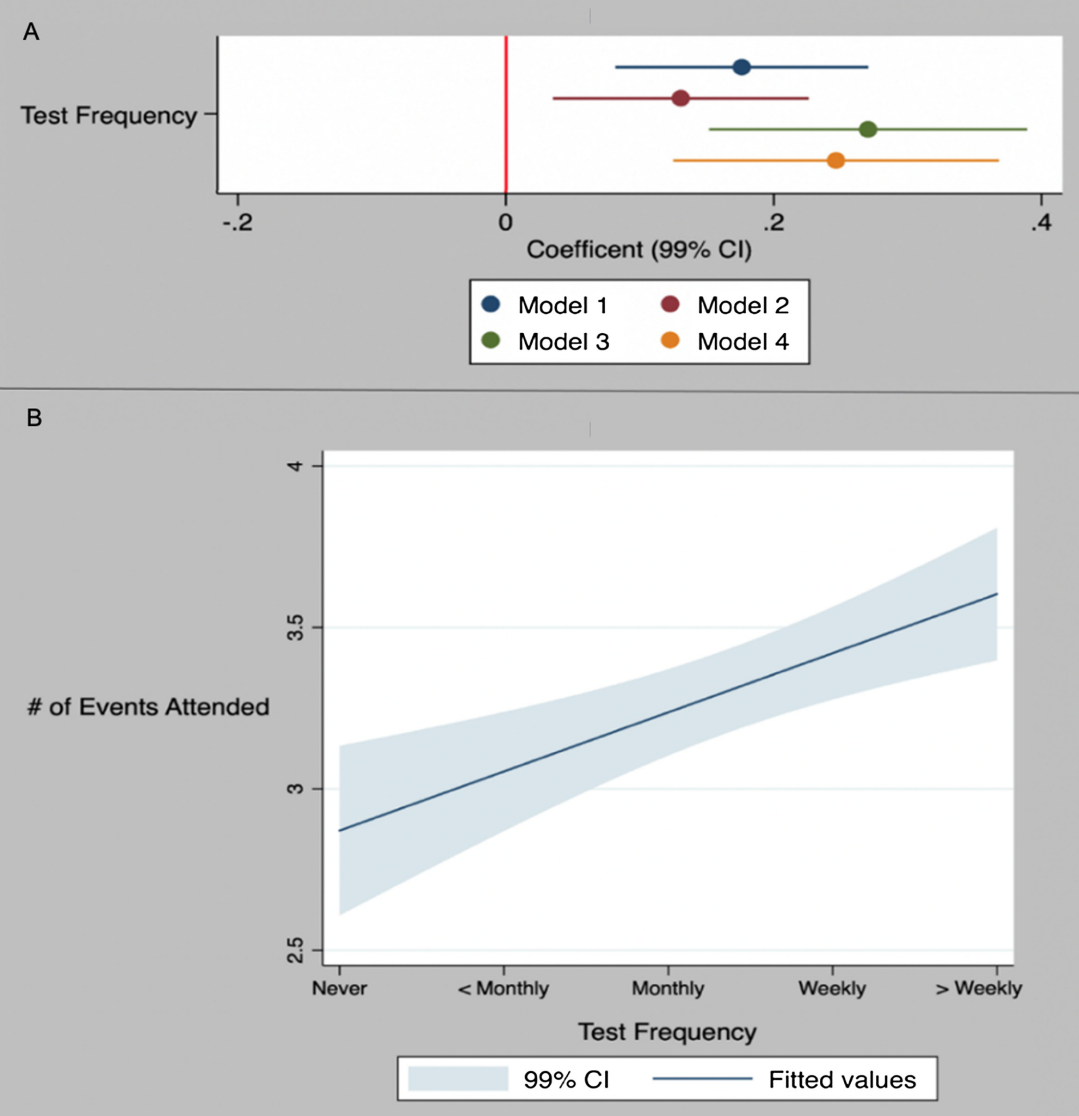
Relationship between COVID-19 test frequency and number of risky events attended—results from Ordered Logit Regression models (*N* = 1,274). *Note*: Panel A: Models 1 to 4 include different independent variables—Model 1: “Test Frequency”; Model 2: “Test Frequency” and “On-line Courses Only” (1 if enrolled in only online courses, 0 otherwise); Model 3: same as Model 2 and “University” (1 for UW, 0 for UI); Model 4: same as Model 3 and political and religious affiliation, COVID-19 health risk perceptions, general risk preferences, year in school, gender, race, and ethnicity (see the Supplementary Material). Panel B: based on Model 1.

Figure 1 shows that more frequent COVID-19 testing is associated with an increase in the number of risky events that a person attends, indicated by the positive and significant coefficient for “Test Frequency” (a 5-point scale ranging from “Never Tested” to “Tested Two or More Times a Week”). This result is robust (i) across models with various sets of covariates including variables previously found to influence COVID-risky behavior (15, 16), (ii) for both university’s sample separately, and (iii) individually for five of the seven risky activities recorded in our study. This result suggests that people exhibit the risk compensation behavior predicted by theory (2). Underlying this finding, respondents who participated in their testing program indicated they perceived the program decreased their risk of contracting COVID-19 (see the Supplementary Material; Wilcoxon Rank Sum, *P* < 0.001), and those who were tested more frequently under the testing program perceived a larger reduction in their risk of contraction with the program than those who were tested less frequently (Ordered Logit, *P* < 0.001). Also, respondents who perceived a larger reduction in the risk of contracting COVID-19 with the testing policy, more frequently attended risky events than those who perceived less risk reduction (Ordered Logit, *P* = 0.016). Overall, results suggest that students perceived that the mandatory testing policy decreased their risk of contracting COVID-19, and that this reduced risk led to higher participation in COVID-risky events.

Consistent with previous research (16, 17), we find women perceive higher health risks from COVID-19 than men, which is robust for the entire sample, as well as university-specific samples (Wilcoxon Rank Sum, *P* < 0.001 for entire sample; *P* = 0.002 for UW; *P* = 0.013 for UI). Also, women are more likely to comply with public policies and follow health recommendations (16), which could indicate a higher trust in the effectiveness of policies and/or a perception that the virus poses a greater health risk. We find women perceive that increased testing reduces the risk of contracting the virus more than men (Wilcoxon Rank Sum, *P* < 0.001, *P* = 0.015, *P* = 0.009 for entire sample, UW, and UI, respectively). These results provide compelling evidence for a gender difference in the behavioral responses to mandatory testing.

**Fig. 2.**
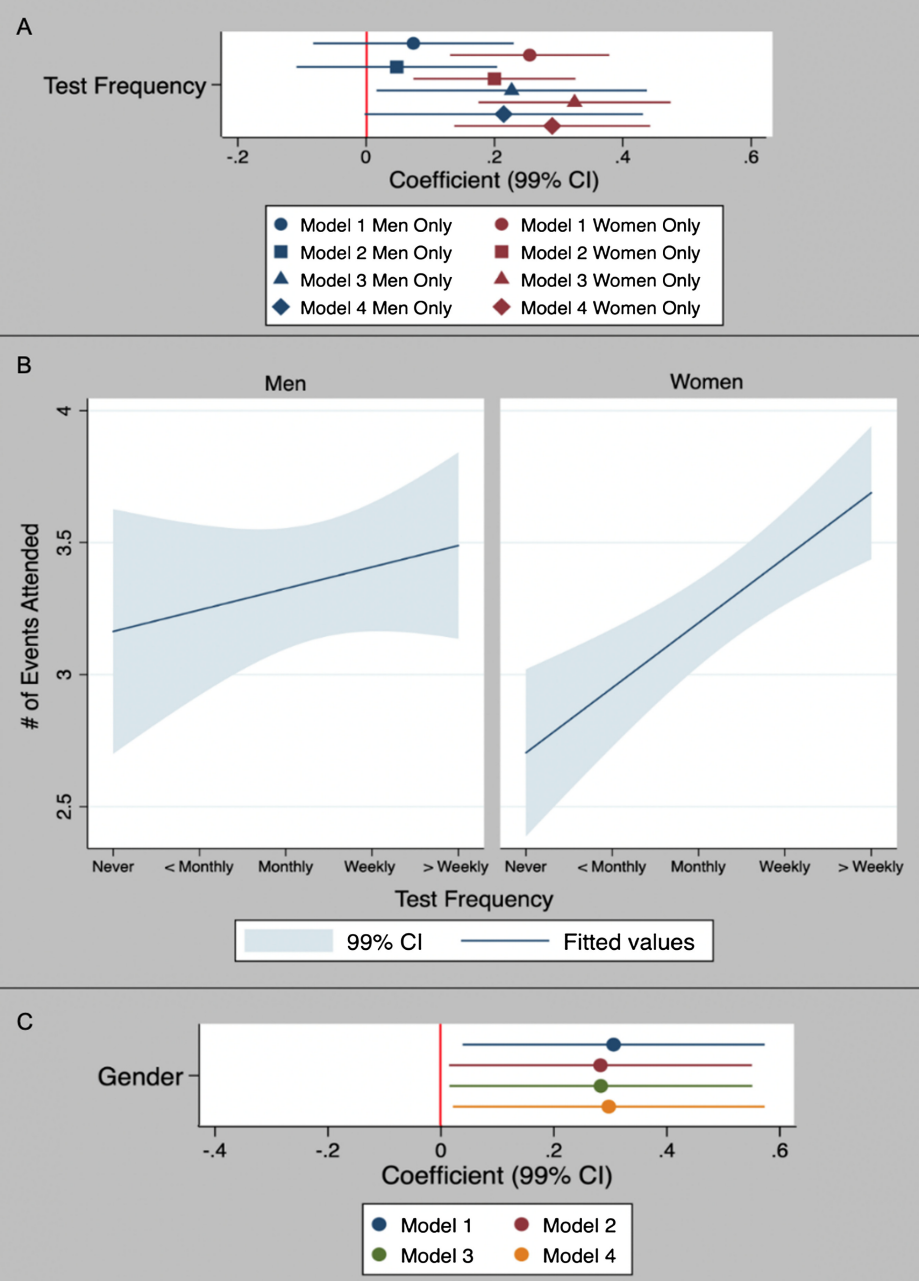
Relationship between COVID-19 test frequency and number of risky events attended by gender—results from Ordered Logit Regression models (*N* = 1,254). *Note*: Panel A: Models 1 to 4 include the same covariates as corresponding models in Fig. 1, excluding gender. Panel B: results from Model 1 (Panel A). Panel C: estimated gender (Woman = 1, Man = 0) coefficient on the magnitude of the relationship between test frequency and risky behavior (see the Supplementary Material) when sample is pooled. Model 1 includes gender as the only independent variable, and Models 2 to 4 include the same covariates as Models 2 to 4 in Fig. 1.

To examine whether there is a gender difference in the relationship between risky behavior and test frequency, we estimate gender-specific regression models for women and men (Fig. 2). [Participants identifying as “genderqueer/gender nonconforming”/“other” (*N* = 20) were excluded.] Results suggest the relationship between participation in risky activities and more frequent testing is stronger for women than men, as indicated by (i) the relationship only being statistically significant for men in Model 3, while always significant for women (Fig. 2, Panel A), and (ii) women exhibiting a stronger relationship than men (Wald test *P* = 0.047, Panel B). To examine the robustness of this gender difference, we estimated the effect that gender has on the magnitude of the relationship between testing and risky behavior (see the Supplementary Material). Panel C, Fig. 2, shows the gender coefficient is positive and statistically significant across all four models, providing additional evidence that the relationship between risky behavior and test frequency is stronger for women. To explore factors that might explain the gender difference, we use mediation analysis of categorical variables (18) and find that differences in the perceived health risk from COVID-19 may partly explain the gender effect (*P* = 0.015).

## Discussion

Our findings suggest that programs with frequent testing may unintentionally increase behavior known to contribute to virus spread. The potential consequences of which are amplified by the exponential nature of viral spread. Thus, when implementing mandatory testing programs to manage a pathogen, it is important to communicate the programs’ limitations in protecting against infection and highlight the potential for unintended behavioral responses. Further, results show the importance of understanding heterogeneity in unintended behavior. If heterogeneous effects exist, public messaging aimed to combat the adverse behavior from policies could be tailored to the groups most prone to engage in increased risky behaviors.

While our results are consistent with risk compensation theory, other explanations to our findings may exist. We address two leading candidates—e.g. self-selection and reverse causality. For self-selection, both campuses announced final testing policies after the start of the fall semester, so it is unlikely students self-selected to either university because of the different testing policies. (UW and UI announced final testing policies on September 1 and September 9.) Turning to reverse causality, if participation in risky events caused an increase in testing, we would expect our results to be particularly strong for students who tested more often than required by their university’s policy. However, our results remain robust if we only consider students whose test frequency matched their university’s testing policy (Ordered Logit, UW: *P* = 0.004; UI: *P* = 0.024). For these reasons, along with the robustness of the results, we have confidence that the findings provide meaningful insights. We encourage future research to corroborate our findings and explore how alternative designs of testing policies can mitigate unintended consequences.

## Materials and methods

Survey participants (*N* = 1,274; *N* = 757 from UW and *N* = 517 from UI) indicated how often they were tested, and how many risky activities they participated in a typical week during the fall semester 2020: large indoor gatherings (10 or more people), small indoor gatherings (four to nine people), and restaurants, both on and off campus; and attended bars off campus. Our main outcome variable is an aggregate measure of risky behavior that defines participation in each risky activity as binary ( =1 if participated in the activity at least once during the semester; 0 otherwise) and then sums participation across the seven activities (ranging between 0 and 7). To ensure high recollection of testing and behavior, we conducted the survey the week immediately following the end of the semester. The survey was approved by each university’s IRB (UW #20201215CR02905, UI #20–209). Participants were recruited through email, consented to participate, and received a $10 gift card upon survey completion.

## Supplementary Material

pgac247_Supplemental_File

## Data Availability

All survey data, data documentation, and code used in the analyses are available at https://doi.org/10.3886/E168241V3.
