# Supplementary material for: Frequent mandatory COVID-19 testing may increase risky behavior
Source: PNAS Nexus. 2022 Nov 4;1(5):pgac247. doi: 10.1093/pnasnexus/pgac247 (PMC9802369; doi:10.1093/pnasnexus/pgac247)
Supplement: pgac247_Supplemental_File [file pgac247_supplemental_file.docx]

**
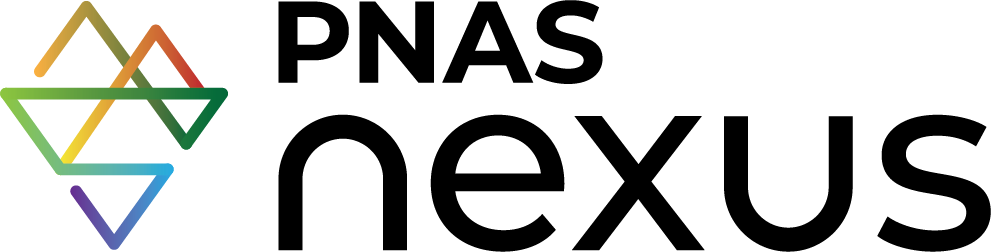
**

**Supplementary Information for**

Frequent mandatory COVID-19 testing may increase risky behavior

Chian Jones Ritten*, Linda Thunström, Todd Cherry, J.D. Wulfhorst

*Chian Jones Ritten

Email: Chian.JonesRitten@uwyo.edu

**This PDF file includes:**

Supplementary text

**Supplementary Information Text**

This Supplemental Information contains details about the survey, coding of data, references, and the survey instrument.

Survey

The survey was administered at UW and UI (N=1,274; N=757 from UW and N=517 from UI) during the week immediately following the end of the Fall 2020 semester. During this semester, the two universities had differing testing policies, but were similar regarding other COVID-related policies. Both universities required mask wearing in all indoor spaces but did not place other specific requirements on activities or gatherings, such as negative tests.

The survey was divided into four sections. In the first section, respondents were asked how often they dined-in at eateries, attended large indoor gatherings (with 10 or more people), attended small indoor gatherings (with between 4 and 9 people), both on and off campus; and how often they drank-in at a bar or club off campus during the fall semester. To limit the influence of the Thanksgiving holiday, respondents indicated if they participated in these events ‘three or more times per week’, ‘about two times per week’, ‘about one time per week’, ‘less than one time per week’, or ‘never’ *on average* over the entire 2020 semester. The second section of the survey asked respondents how often they were tested on average (‘two or more times a week’, ‘one time a week’, ‘one time a month’, ‘less than one time a month’, or ‘never was tested’). The third section asked respondents about their perceived risk associated with COVID-19. The last section asked about their mode of coursework (online or in-person), demographics, political orientation, religious beliefs, and general risk preferences.

Data Coding

Since there is a relatively high prevalence of respondents indicating that they never engaged in risky activities, we recoded the frequency of attending events for each activity as a dichotomous variable for regression analyses. Therefore, for each activity (eating-in at eateries, attending small and large gatherings (both on and off campus), and drinking-in at bars or clubs off campus), the corresponding variable was coded as 0 if the respondent reported never attending the event, and 1 otherwise. The number of 1s for these dichotomous variables where then aggregated across activities for each respondent to create a count variable that indicates the number of these events that the respondent attended (ranging from 0 if the respondent never attended any of these events to 7 if the respondent attended all of these events at least one time). The resulting categorical variable, Number of Events Attended, is used as the dependent variable in Models 1-4 reported in Fig 1 and in Panels A and B in Fig 2.

The variable, Test Frequency, was coded as a categorical variable based on respondents’ responses to how often they were tested, on average, during the semester (‘two or more times a week’ coded as 5, ‘one time a week’ coded as 4, ‘one time a month’ coded as 3, ‘less than one time a month’ coded as 2, or ‘never was tested’ coded as 1). Test Frequency was used as the primary independent variable in the ordered logit regressions reported in Panel A of Fig 1 and 2 and the trends in Panel B of Fig 1 and 2.

Respondents were also asked to indicate their political and religious affiliation in the survey. A dummy variable was created indicating whether a respondent indicated they identified as liberal or not. This variable was coded as 1 when a respondent indicated they were either ‘very liberal’ or ‘somewhat liberal’ and coded as 0 when a respondent indicated they were ‘moderate’, ‘somewhat conservative’, or ‘very conservative’. Because the most commonly identified religious affiliation was Christianity, a dummy variable was coded as 1 if a respondent identified as Christian, and 0 otherwise. Both of these dummy variables were used as covariates in Model 4 of Fig 1 and 2.

Respondents were asked multiple questions regarding their perceptions of their personal risks associated with contracting, and the resulting sickness from, SARS-CoV-2. Specifically, respondents were asked, how likely is it that ‘you will be diagnosed with COVID-19 within the next month’; how likely is it that ‘if diagnosed, it would limit your daily activities’; how likely is it that ‘if diagnosed, it would lead to your hospitalization’; and how likely is it that ‘if diagnosed, it would lead to your death’ (‘very unlikely’ coded as 1, ‘unlikely’ coded as 2, ‘moderately likely’ coded as 3, ‘likely’ coded as 4, and ‘very likely’ coded as 5). Respondents were also asked to indicate, ‘compared to other people your age, how likely are you to be diagnosed with COVID-19’ and ‘compared to other people your age, what is the likelihood of you being hospitalized if diagnosed with COVID-19’ (‘much less likely’ coded as 1, ‘less likely’ coded as 2, ‘about the same’ coded as 3, ‘more likely’ coded as 4, and ‘much more likely’ coded as 5). The responses to these 6 questions were aggregated for each individual to create a new variable that measures general COVID-19 risk perceptions that ranges from 5 to 30, with smaller values indicating lower risk perceptions, and higher values indicating higher risk perceptions. This aggregate risk perception variable was used as a covariate in Model 4 of Fig 1 and 2. Additionally, this variable was tested to determine if it acts as a mediation variable for the gender difference found in the effect of mandatory surveillance on risky behavior.

General risk preferences were also collected following Eckel and Grossman [1]. A respondent’s year in school (‘Freshman’, ‘Sophomore’, ‘Junior’, ‘Senior’, or ‘Graduate Student’), race (identify as ‘White’ or ‘Non-White’), and ethnicity (identify as ‘of Hispanic, Latino, or Spanish Origin’ or not) were also collected. These variables were included in Model 4 reported in Fig 1 and 2.

Respondents were asked if they believed their university’s testing program affected their risk of contracting the coronavirus both on and off campus (increased their risk coded as 1, caused no change in risk coded as 2, and decreased their risk coded as 3). Responses to these two question (on and off campus) were then aggregated to generate a variable that measured how respondents’ perceived risk of contracting COVID-19 changed in response to their testing program (values ranging from 2 to 6). This variable was used to determine whether participating in their university’s testing program, and how often they were tested, affected respondent’s believed risk of contracting the virus, and how that perceived risk influenced the number of risky events respondents attended.

To explore the robustness of the gender difference in the relationship between test frequency and risky activities observed in our study, an individual’s magnitude of this relationship was determined (ranging from 0 to 3) based on the individual’s Test Frequency and Number of Events Attended. Respondents with low (high) Test Frequency and low (high) Number of Events Attended were assigned a value of 3 for this relationship variable. Respondents with low (high) Test Frequency and high (low) Number of Events Attended were assigned a value of 0 for the relationship variable. Respondents with a relatively low (high) Test Frequency and relatively low (high) Number of Events Attended were assigned a value of 2 for the relationship variable, while respondents with a relatively low (high) Test Frequency and relatively high (low) Number of Events Attended were assigned a value of 1 for the relationship variable. Respondents with a higher value for this variable have a have a stronger positive relationship between test frequency and risky activities than those individuals with lower values. The resulting variable was used as the dependent variable in Panel C of Fig 2.

**Survey Instrument**

Welcome to our study! The survey is expected to last 15 minutes. Please note that there are no "right" or "wrong" answers. We ask you to answer all questions as truthfully as possible. You must be 18 years or older and a current University of Wyoming (University of Idaho) student to complete the survey. Please note that you can only complete the survey one time.

Thank you for your participation in this study. We highly value your contribution to our project. Before you start the survey, we need your consent to participate. Please read the following statement:

I have been selected to participate in a survey about the coronavirus, which is expected to last 10-15 minutes. No data will be released from my survey where my individual identity can be determined. Data will be stored in computer files for the duration of the research project, accessible only to the primary and co-investigators involved in the research project.

I have not been requested to waive or release the institution, its agents or sponsors, from liability for the negligence of its agents or employees. My participation is voluntary, and I may discontinue participation at any time. If I have any questions or wish to obtain more information regarding this research or my rights as a participant, I can contact Chian Jones Ritten at: chian.jonesritten@uwyo.edu, 766-3788, Ag Bldg. Room 216D.

I have read the above information and agree to participate in the survey.

- Yes
- No -- If selected, you will not be able to continue with the survey

PART 1. We begin by asking about your situation at the University of Wyoming (University of Idaho).

Were you a student at the University of Wyoming: hereafter UW (University of Idaho: hereafter UI) in the fall semester of 2020?

- Yes
- No

Which of the following best characterizes how often you were on campus during the fall semester?

- Daily
- Every other day
- Once a week
- Every other week
- Monthly
- Less than monthly
- Never

Approximately how many times during the fall semester did you participate in the following on-campus activities?

|  | Never | Less than one (1) time per week | About one (1) time per week | About two (2) times per week | Three (3) or more times per week |
| --- | --- | --- | --- | --- | --- |
| Dine-in at eateries on-campus |  |  |  |  |  |
| Large indoor gatherings on-campus (10 or more people |  |  |  |  |  |
| Small indoor gatherings on-campus (4 to 9 people) |  |  |  |  |  |

Approximately how many times during the fall semester did you participate in the following off-campus activities?

|  | Never | Less than one (1) time per week | About one (1) time per week | About two (2) times per week | Three (3) or more times per week |
| --- | --- | --- | --- | --- | --- |
| Dine in at restaurants – off-campus |  |  |  |  |  |
| Drink-in at a bar/club – off-campus |  |  |  |  |  |
| Large indoor gatherings off-campus (10 or more people) |  |  |  |  |  |
| Small indoor gatherings off-campus (4 to 9 people) |  |  |  |  |  |

Which of the following best characterize your attendance at indoor social events during the semester?

- I attended MORE events towards the end of the semester than the beginning of the semester
- I attended FEWER events towards the end of the semester than the beginning of the semester
- I attended about the SAME number of events throughout the semester

PART 2. The next set of questions focus on your experiences with COVID-related university policies during the fall semester.

Were you required to participate in the COVID-19 testing program while on campus at UW (UI) during the fall semester?

- Yes
- No

How many COVID-19 tests per week/month did you take in a typical month while on campus during the fall semester?

- Two (2) or more tests a week
- One (1) test a week
- One (1) test a month
- Less than (1) test a month
- I never got a test

Now, imagine that UW (UI) had no testing program and did not require you (or anyone else in the UW (UI) community) to get tested during the semester. Also, assume you could take a test at a nearby facility, at no financial cost to you. Under those conditions, how many COVID-19 tests per week/month do you think you would have taken while on campus during the fall semester?

- Two (2) or more tests a week
- One (1) test a week
- One (1) test a month
- Less than (1) test a month
- I would never get a test

Was there ever a time during the fall semester at UW (UI) that you wanted to be tested but could not get a test?

- Yes
- No

Regarding the UW (UI) testing program, how would you characterize the extent of the testing program during the fall semester?

- Comprehensive -- 90% or more of people on campus were regularly tested
- Widespread -- 70-89% of people on campus were regularly tested
- Partial -- 50-69% of people on campus were regularly tested
- Limited -- 30-49% of people on campus were regularly tested
- Scarce -- less than 30% of people on campus were regularly tested

Focusing on the UW (UI) testing program, how did the following things change for you because of the UW (UI) testing program (compared to having no testing program)?

|  | Increased | Decreased | No change |
| --- | --- | --- | --- |
| Your risk of contracting the coronavirus on campus |  |  |  |
| Your risk of contracting the coronavirus off campus |  |  |  |
| Your willingness to attend indoor social events on campus |  |  |  |
| Your willingness to attend indoor social events off campus |  |  |  |

Regarding the UW (UI) mask mandate, how would you characterize the general campus compliance with the mask mandate during the fall semester?

- Comprehensive -- 90% or more of people on campus wore a mask
- Widespread -- 70-89% of people on campus wore a mask
- Partial -- 50-69% of people on campus wore a mask
- Limited -- 30-49% of people on campus wore a mask
- Scarce -- less than 30% of people on campus wore a mask

Regarding the UW (UI) mask mandate, how would you characterize your compliance with the mask mandate during the fall semester?

- Comprehensive -- I wore a mask 90% or more of the time
- Widespread -- I wore a mask 70-89% of the time
- Partial -- I wore a mask 50-69% of the time
- Limited -- I wore a mask 30-49% of the time
- Scarce -- I wore a mask less than 30% of the time

Focusing on the UW (UI) mask-wearing mandate, how did the following things change for you because of the UW (UI) mask mandate (compared to having no mandate)?

|  | Increased | Decreased | No change |
| --- | --- | --- | --- |
| Your risk of contracting the coronavirus on campus |  |  |  |
| Your risk of contracting the coronavirus off campus |  |  |  |
| Your willingness to attend indoor social events on campus |  |  |  |
| Your willingness to attend indoor social events off campus |  |  |  |

How did the UW (UI) testing program change your decisions to wear facemasks at indoor social events off-campus?

- It made me wear facemasks MORE frequently
- It made me wear facemasks LESS frequently
- It had no effect on how often I wore facemask

PART 3. The next few questions focus on your plans during the winter break.

During the winter break, how often do you plan to attend the following indoor social events?

|  | Never | Less than once per week | About one (1) time per week | About two (2) times per week | Three (3) or more times per week |
| --- | --- | --- | --- | --- | --- |
| Dine in at restaurants |  |  |  |  |  |
| Drink-in at a bar/club |  |  |  |  |  |
| Large indoor gatherings (10 or more people) |  |  |  |  |  |
| Small indoor gatherings (4 to 9 people) |  |  |  |  |  |

During the winter break, how often do you plan to do the following?

|  | Never | Some of the time | Most of the time | All of the time | Not applicable |
| --- | --- | --- | --- | --- | --- |
| Wear a facemask in public spaces |  |  |  |  |  |
| Keep social distance when in public |  |  |  |  |  |

Have you taken a COVID-test since you have left UW (UI) campus?

- Yes, 3 or more times
- Yes, 2 times
- Yes, 1 time
- No, not since leaving campus for winter break

Under what circumstances would you take a COVID-19 test during winter break?

- I plan on taking a COVID-19 test even if I do not have any symptoms or have not been in contact with someone who is diagnosed with the coronavirus
- I would take a COVID-19 test but only if I have symptoms or have been in contact with someone who is diagnosed with the coronavirus
- I will not take a test under any circumstance

PART 4. The next set of questions focus on your opinions on COVID-related issues.

What is your general opinion of the federal government’s actions to limit the spread of the coronavirus?

- The government has intervened far too much
- The government has intervened a bit too much
- The government has intervened about the right amount
- The government has intervened a bit too little
- The government has intervened far too little

What is your general opinion of the U.S. population getting an FDA approved vaccine for COVID-19 when available?

- Everyone in the U.S. should be required to vaccinate, unless they have a medical excuse
- Everyone in the U.S. should be recommended to vaccinate
- No one in the U.S. should be recommended to vaccinate
- Everyone in the U.S. should be discouraged to vaccinate

What is your general opinion of UW (UI) students, staff and faculty getting an FDA approved vaccine for COVID-19 when available?

- Everyone at UW (UI) should be required to vaccinate, unless they have a medical excuse
- Everyone at UW (UI) should be recommended to vaccinate
- No one at UW (UI) should be recommended to vaccinate
- Everyone at UW (UI) should be discouraged to vaccinate

Do you support the continuation of the UW (UI) testing program during the spring semester (the same program that was in place during the fall semester)?

- Yes
- No

Do you support the continuation of UW’s (UI’s) face mask requirements during the spring semester (the same face mask requirements that were in place during the fall semester)?

- Yes
- No

PART 5. The next set of questions asks about your experience with COVID-19.

Did you have a positive COVID-19 test during the fall semester (i.e., did any test indicate that you were infected with the coronavirus)?

- Yes
- No

Do you personally know anyone that tested positive for COVID-19 during the fall semester?

- Yes
- No

Do you personally know anyone who has been hospitalized because of COVID-19?

- Yes
- No

Do you personally know anyone who has died from COVID-19?

- Yes
- No

Do you live or work with someone who is more vulnerable to having complications from COVID-19?

- Yes
- No

Please indicate how likely you believe it is that each of the following will happen to you

|  | Very unlikely | Unlikely | Moderately likely | Likely | Very likely |
| --- | --- | --- | --- | --- | --- |
| You will be diagnosed with COVID-19 within the next month |  |  |  |  |  |
| If diagnosed, it would limit your daily activities |  |  |  |  |  |
| If diagnosed, it would lead to your hospitalization |  |  |  |  |  |
| If diagnosed, it would lead to your death |  |  |  |  |  |

Compared to other people your age in the U.S., what is the likelihood of you being diagnosed with COVID-19?

- Much more likely
- More likely
- About the same
- Less likely
- Much less likely

Compared to other people your age in the U.S., what is the likelihood of you being seriously ill (i.e., hospitalized) if diagnosed with COVID-19?

- Much more likely
- More likely
- About the same
- Less likely
- Much less likely

Please indicate your level of agreement with the following statements

|  | Strongly agree | Agree | Neither agree nor disagree | Disagree | Strongly disagree |
| --- | --- | --- | --- | --- | --- |
| If I wear a mask, the risk of me contracting the coronavirus decreases |  |  |  |  |  |
| If I wear a mask, the risk of me spreading the coronavirus to others decreases |  |  |  |  |  |
| If I am tested for COVID regularly, the risk of me contracting the coronavirus decreases |  |  |  |  |  |
| If I am tested for COVID-19 regularly, the risk of me spreading the coronavirus to others decreases |  |  |  |  |  |

PART 6. The next set of questions ask some background information that allows us to ensure results are representative.  As mentioned, responses are anonymous and cannot be linked to any individual.

Which of the following best describes you? I am a...

- Freshman
- Sophomore
- Junior
- Senior
- Graduate student
- Other

In which college at UW (UI) is your major located?

- College of Agriculture and Natural Resources
- College of Arts and Sciences
- College of Business
- College of Education
- College of Engineering and Applied Science
- College of Health Sciences
- College of Law
- Haub School of Environment and Natural Resources
- None - I do not have a major
- I don't know

What was the delivery mode of your UW (UI) classes during the fall semester?

- All my classes were in-person
- Some of my classes were in-person and some classes were online
- All my classes were online

What was your enrollment status during the fall semester?

- Full time
- Part time
- Other

What is your current gender identity?

- Male/man
- Female/women
- Trans male/Trans man
- Trans female/Trans woman
- Genderqueer/Gender non-conforming
- Other

Please indicate your race (check all that apply).

- White
- Black or African American
- American Indian or Alaska Native
- Asian
- Native Hawaiian or Pacific Islander
- Other

Are you of Hispanic, Latino, or Spanish origin? (Check most appropriate category)

- Yes, Mexican, Mexican American, Chicano, Puerto Rican, Cuban, or other Hispanic, Latino, or Spanish origin
- No, not of Hispanic, Latino, or Spanish origin

In what year were you born?

________________________________________________________________

What is the zip code of where you currently reside?

________________________________________________________________

How many adults did you live with during the part of the fall semester when students were on campus?

- 3 or more
- 2
- 1
- 0

How many children did you live with during the part of the fall semester when students were on campus?

- 3 or mor
- 2
- 1
- 0

What is your individual total annual income (pretax)?

- Less than $10,000
- $10,001 – 20,000
- $20,001 – 30,000
- $30,001 – 50,000
- $50,001 – 100,000
- $101,000 and above
- I do not know

What is your political orientation?

- Very liberal
- Somewhat liberal
- Moderate
- Somewhat conservative
- Very conservative

What is your religious preference?

- Jewish
- Muslim
- Christian
- Buddhist
- Hindu
- None -- I identify with atheism
- None -- I identify with agnosticism
- Other

Please indicate your level of agreement with the following statements

|  | Strongly agree | Agree | Somewhat agree | Neither agree nor disagree | Somewhat disagree | Disagree | Strongly disagree |
| --- | --- | --- | --- | --- | --- | --- | --- |
| The federal government should declare the United States a Christian nation |  |  |  |  |  |  |  |
| The federal government should advocate Christian values |  |  |  |  |  |  |  |
| The federal government should allow prayer in public schools |  |  |  |  |  |  |  |

Imagine that you have to participate in one gamble from the six different gambles below. Each gamble has two possible outcomes ("high outcome" and "low outcome"). Each outcome has a 50% chance of occurring. For instance, for Gamble 4, the "high outcome" of $52 has a 50% chance of occurring, and the "low outcome" of $16 also has a 50% chance of occurring. 


Please select the gamble below that you would have preferred to participate in.

- Gamble 1- low outcome = $28, high outcome = $28
- Gamble 2- low outcome = $24, high outcome = $36
- Gamble 3- low outcome = $20, high outcome = $44
- Gamble 4- low outcome = $16, high outcome = $52
- Gamble 5- low outcome = $12, high outcome = $60
- Gamble 6- low outcome = $2, high outcome = $70

**SI References**

1. C. C. Eckel, P. J. Grossman. Sex differences and statistical stereotyping in attitudes toward financial risk. *Evol. Hum. Behav.* 23(4), 281– 95 (2002).
